# Modeling and therapeutic targeting of t(8;21) AML with/without *TP53* deficiency

**DOI:** 10.1007/s12185-024-03783-3

**Published:** 2024-05-03

**Authors:** Wenyu Zhang, Jingmei Li, Keita Yamamoto, Susumu Goyama

**Affiliations:** Division of Molecular Oncology, Department of Computational Biology and Medical Sciences, Graduate School of Frontier Sciences, The University of Tokyo, 4-6-1 Shirokanedai, Minato-ku, Tokyo, 108-8639 Japan

**Keywords:** AML, RUNX1-ETO, TP53, CRISPR/Cas9, Mouse model

## Abstract

Acute myeloid leukemia (AML) with t(8;21)(q22;q22.1);RUNX1-ETO is one of the most common subtypes of AML. Although t(8;21) AML has been classified as favorable-risk, only about half of patients are cured with current therapies. Several genetic abnormalities, including *TP53* mutations and deletions, negatively impact survival in t(8;21) AML. In this study, we established Cas9^+^ mouse models of t(8;21) AML with intact or deficient *Tpr53* (a mouse homolog of *TP53*) using a retrovirus-mediated gene transfer and transplantation system. *Trp53* deficiency accelerates the in vivo development of AML driven by RUNX1-ETO9a, a short isoform of RUNX1-ETO with strong leukemogenic potential. *Trp53* deficiency also confers resistance to genetic depletion of RUNX1 and a TP53-activating drug in t(8;21) AML. However, *Trp53*-deficient t(8;21) AML cells were still sensitive to several drugs such as dexamethasone. Cas9^+^ RUNX1-ETO9a cells with/without *Trp53* deficiency can produce AML in vivo, can be cultured in vitro for several weeks, and allow efficient gene depletion using the CRISPR/Cas9 system, providing useful tools to advance our understanding of t(8;21) AML.

## Introduction

The t(8;21)(q22;q22) translocation resulting in the RUNX1-ETO (also called AML1-ETO or RUNX1-RUNX1T1) rearrangement is one of the most common genetic alterations in acute myeloid leukemia (AML), accounting for 5–10% of AML cases [1–3]. Patients diagnosed with t(8;21) AML typically experience a favorable prognosis when treated with intensive cytarabine-based chemotherapy. However, a significant number of patients still relapse, highlighting the clinical heterogeneity within t(8;21) AML [4, 5]. Relapse rates are particularly high in elderly patients who are unable to tolerate high-dose cytotoxic chemotherapy.

RUNX1-ETO alone is not sufficient for leukemogenic transformation and requires additional genetic alterations for progression to full blown AML [1, 3]. Studies have identified the collaborative genetic alterations, including mutations in *KIT*, *ASXL1*, *ZBTB7A*, *NRAS*, *CBL*, and *TP53* genes in t(8;21) AML [5, 6]. Some of these mutations, such as those in *KIT*, *ASXL1*, and *TP53*, negatively affect survival. In particular, loss of the TP53 response pathway has been shown to be associated with drug resistance and disease progression in RUNX1-ETO leukemia [7], while RUNX1-ETO itself was shown to activate the p53 pathway that sensitizes leukemia cells to DNA damage [8]. Therefore, new approaches for the treatment of t(8;21) AML patients with *TP53* mutations or deletions need to be developed.

In addition to the full-length RUNX1-ETO, alternatively spliced isoforms of the RUNX1-ETO transcript have been identified in t(8;21) patients. RUNX1-ETO9a [9], a short isoform of RUNX1-ETO, encodes a C-terminally truncated RUNX1-ETO protein with a stronger leukemogenic potential than full-length RUNX1-ETO. Because RUNX1-ETO9a can induce AML without cooperating mutations in a mouse retroviral transduction-transplantation model, it has been widely used experimentally as mouse models of t(8;21) AML.

In this study, we developed novel mouse models for t(8;21) AML using RUNX1-ETO9a, *Trp53* (the mouse homolog of *TP53*)-deficient mice and Cas9 knockin mice. The established Cas9^+^, RUNX1-ETO9a-expressing AML cells with/without *Trp53* deficiency will be useful tools for the development of effective therapeutic strategies for t(8;21) AML.

## Methods

### Mice

C57BL/6 mice (Ly5.1) obtained from Sankyo Labo Service Corporation, Tokyo, Japan, were employed in bone marrow transplantation assays. *Trp53*^−/−^ mice were sourced from the RIKEN BioResource Center in Ibaragi, Japan [10]. Rosa26-LSL-Cas9 knockin mice were procured from The Jackson Laboratory (#024857) [11]. To generate *Trp53*^−/−^-Cas9 mice, *Trp53*^−/−^ mice were bred with Cas9 knockin mice. All animal experiments were granted approval by the Animal Care Committee of the Institute of Medical Science at the University of Tokyo (PA21-67) and were carried out in accordance with the Regulation on Animal Experimentation at the University of Tokyo, following the International Guiding Principles for Biomedical Research Involving Animals.

### Cell culture

RUNX1-ETO9a-Cas9^+^ and RUNX1-ETO9a-*Trp53*^*−/−*^*-*Cas9^+^ cells were isolated from spleens of leukemic mice. Initially, these cells were cultured in Roswell Park Memorial Institute (RMPI)-1640 medium (#189–02025, FUJIFILM Wako) supplemented with 10% fetal bovine serum (FBS; #FB-1365/500, Biosera), and 1% penicillin–streptomycin (PS, #09367–34, Nacalai), along with the following cytokines: 100 ng/ml SCF (#455-MC, R&D Systems), 10 ng/ml IL-6 (#216–16, PEPROTECH), and 1 ng/ml IL-3 (#213–13, PEPROTECH). The amount of cytokines was gradually reduced, and eventually the cells were maintained in the same medium supplemented only with 1 ng/ml IL-3. When the cryopreserved cells were used, the cells were initially cultured with 100 ng/ml SCF, 10 ng/ml IL-6 and 1 ng/ml IL-3 for one week and then cultured with 1 ng/ml IL-3 only.

cSAM cells were previously generated using a murine transplantation model. Briefly, a C-terminally truncated form of ASXL1 and SETBP-D868N were transduced into mouse bone marrow progenitor cells, and transplanted into sublethally irradiated recipient mice. Leukemic cells were isolated from the bone marrow of the moribund mice and their leukemogenic activity was confirmed by serial transplantation [12, 13]. The cSAM cells were cultured in RPMI-1640 medium supplemented with 10% FBS and 1 ng/ml IL-3.

Plat-E [14] and 293 T cells were cultured in Dulbecco's Modified Eagle Medium (DMEM) medium (#044–29765, Wako) with 10% FBS and 1% PS.

### Plasmids and Viral transduction

HA-tagged RUNX1-ETO9a was employed for RUNX1-ETO9a expression, and it was incorporated into a pMSCV-IRES-Thy1.1 retroviral vector [15]. Retroviruses were produced by transiently transfecting Plat-E packaging cells using the calcium-phosphate coprecipitation method. The cells were transduced with retroviruses using Retronectin (Takara Bio Inc., Otsu, Shiga, Japan). Lentiviruses were produced by transiently transfecting lentiviral plasmids, along with PCMV-VSV-G (Addgene, #8454) [16] and psPAX2 (Addgene, #12,260), into 293 T cells with the calcium-phosphate method [15].

### Runx1 depletion using the CRISPR/Cas9

To generate single guide RNA (sgRNA), annealed oligos were incorporated into either the pLentiguide-puro vector (#52,962) [17] or pLKO5.sgRNA.EFS.tRFP657 vector (#57,824) [18], both sourced from Addgene. For the stable expression of puromycin-resistant sgRNAs, RUNX1-ETO9a-Cas9^+^ cells were transduced with the sgRNAs and were subsequently selected using puromycin (1 μg/ml) in RPMI-1640 medium with 10% FBS, 1% PS, and 1 ng/ml IL-3. The sequences for the non-targeting (NT) control and sgRNAs targeting mouse *Runx1* are provided below: NT: 5′ cgcttccgcggcccgttcaa 3′, sg*Runx1*-(1): 5′ tgcgcactagctcgccaggg 3′, sg*Runx1*-(2): 5’ agaactgagaaatgctaccg 3′.

### Transplantation assay

5-FU (150 mg/kg, intraperitoneal injection) was administered to male mice carrying the Cas9 gene and *Trp53*^−/−^-Cas9 mice. The bone marrow was collected from the Cas9 mice and *Trp53*^*−/−*^-Cas9 mice after 4 days, and were pre-cultured in RPMI-1640 containing 10% FBS, 1% PS, supplemented with 100 ng/ml murine SCF, 1 ng/ml IL-3 and 10 ng/ml IL-6 for 16 h. These cells were then transduced with RUNX1-ETO9a retrovirus and transplanted into lethally irradiated (9.5 Gy) 8 weeks-old male Ly5.1 mice. Each mouse received 2 × 10^5^ RUNX1-ETO9a-transduced cells with 2 × 10^5^ wild type Ly5.1 bone marrow cells. For serial transplantation, 1 × 10^6^ spleen or bone marrow cells collected from moribund leukemic mice were intravenously injected into sublethally irradiated (5.25 Gy) male Ly5.1 mice. Note that male mice were used as recipient mice in all experiments to avoid a potential immune response of female mice to male donor cells.

### Flow Cytometry

Fluoro-conjugated antibodies were used to stain cells for 15 min at 4 °C. Following staining, cells underwent two cold PBS washes and were then resuspended in PBS containing 2% FBS. Subsequently, analysis of the cells was conducted using Canto II (BD Biosciences, San Jose, CA, USA) and FlowJo software (FlowJo). The antibodies employed in this study are listed below: APC-CD90.1 (Thy1.1), Biolegend; #202,526, BV421-c-kit, Biolegend; #135,123, PE-Cy5-CD11b, Biolegend; # 101,210 and PE-Gr-1, Biolegend; # 127,608. The dilution ratio of these antibodies was 1:400.

### Western blotting

Cells underwent multiple washes with PBS and were then lysed using pre-heated Laemmli sample buffer (Bio-rad, USA; #1,610,737). The resulting total cell lysates were subjected to SDS-PAGE and transferred onto a polyvinylidene fluoride membrane (Bio-Rad). The membrane was incubated with anti-RUNX1 antibody [AML1 (D4A6) Rabbit mAb #8529; Cell Signaling Technology, Beverly, MA, 1:500] and anti-GAPDH antibody [GAPDH (D16H11) XP® Rabbit mAb #5174; Cell Signaling Technology, Beverly, MA, 1:500]. Signals were detected using ECL Western Blotting Substrate (Promega, Madison, WI, USA) and visualized with the LAS‐4000 Luminescent Image Analyzer (FUJIFILM).

### Cell growth assay

The cytotoxic effects of DS-5272, Cytarabine, Dexamethasone, and Decitabine against RUNX1-ETO9a cells with/without Trp53 deficiency or cSAM cells were assessed using the Cell Counting Kit-8 (Dojindo, Kumamoto, Japan) following the manufacturer's instructions. Cells were plated in 96-well plates at a density of 5 × 10^3^ cells/well in 0.1 ml medium and treated with various concentrations of each compound. After 72 h of incubation at 37 °C, 8 μl of the Cell Counting Kit-8 was added to each well. Following a 1-h incubation at 37 °C, the absorbance at 450 nm was measured using a microplate reader (CLARIOstar Plus, BMG LABTECH, Ortenberg, GER). Relative cell viability was expressed as the ratio of the absorbance in each treatment group to that of the corresponding untreated control group. The data are presented as means ± standard deviation (SD) from more than three independent experiments. IC50 values were calculated using GraphPad Prism software.

### Statistical analyses

GraphPad Prism 10 was employed for all statistical analyses. Pairwise comparisons of significance were carried out utilizing ordinary two-way ANOVA. Survival curve comparisons were performed using the log-rank (Mantel-Cox) test. Animal experiments were not subjected to blinding or randomization. Sample sizes were determined based on prior experience rather than a predetermined statistical method. All data are presented as mean ± SD.

## Results

### *Trp53* deficiency accelerates the development of RUNX1-ETO9a-induced AML

**Fig. 1:**
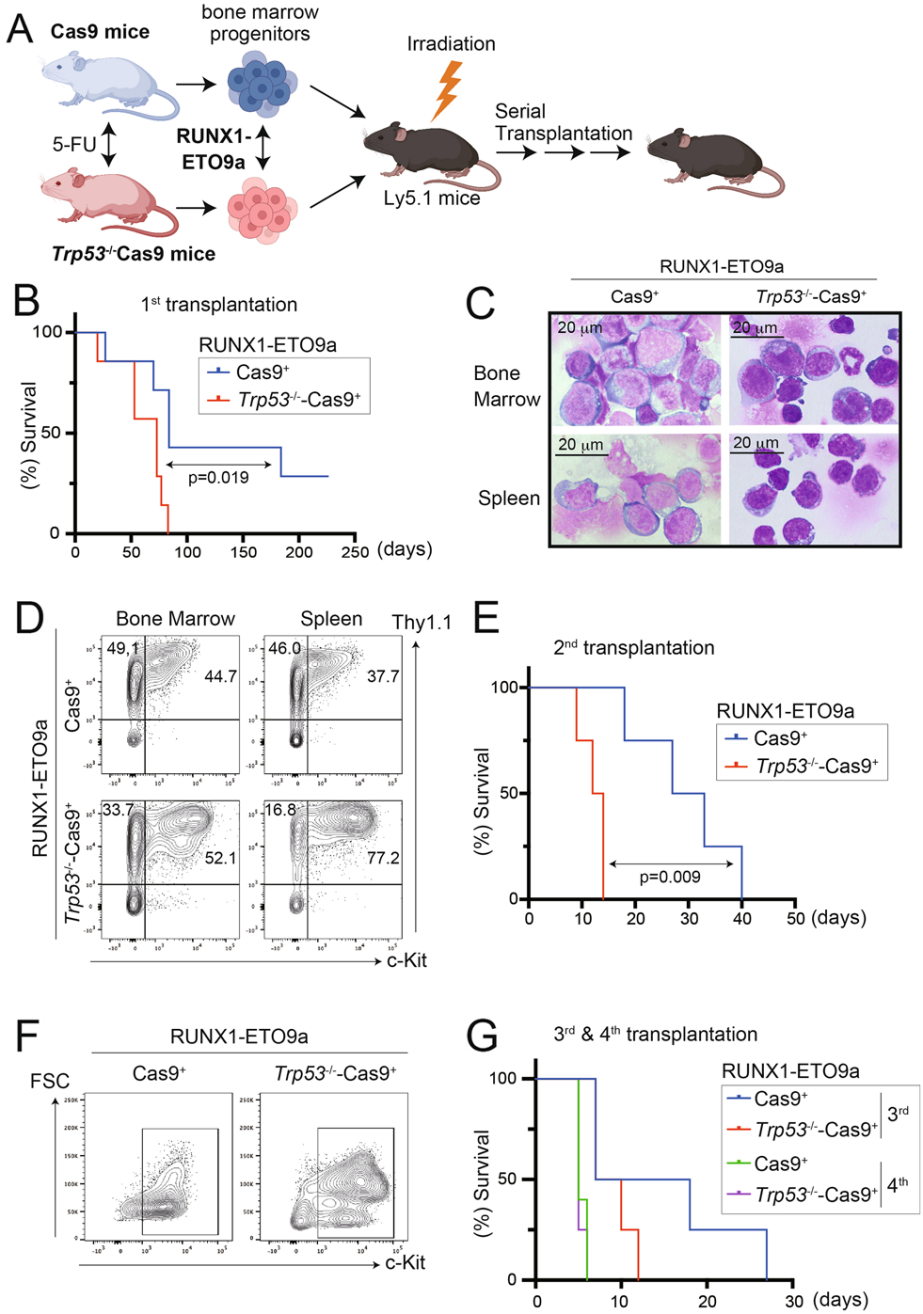
Modeling of RUNX1-ETO leukemia with/without *Trp53* deficiency.** A** Scheme of the experiments used in **B**-**F**. Bone marrow cells were collected from 5-FU-treated Cas9 mice or *Trp53*^*−/−*^-Cas9 mice, transduced with RUNX1-ETO9a, and were transplanted into lethally irradiated recipient mice. Figures were created partly with BioRender (https://app.biorender.com/). **B** Kaplan–Meier survival curves of mice transplanted with RUNX1-ETO9a-Cas9^+^ cells or RUNX1-ETO9a-*Trp53*^−/−^-Cas9^+^ cells are shown (n = 7 each group). **C**, **D** Leukemia cells were collected from spleens and bone marrows of moribund mice transplanted with RUNX1-ETO9a-Cas9^+^ cells or RUNX1-ETO9a-*Trp53*^−/−^-Cas9^+^ cells. These cells were subjected to the Wright-Giemsa staining (**C**, Magnification, × 100) or FACS analysis (**D**). **E** Leukemia cells were collected from spleens or bone marrows of primary recipient mice and were transplanted into secondary recipient mice. Kaplan–Meier survival curves of these mice are shown (n = 4 each group). **F**. Leukemia cells were collected from spleens of moribund secondary mice and subjected to FACS analysis. **G** Tertiary and quaternary transplantations were performed using RUNX1-ETO9a-Cas9^+^ cells or RUNX1-ETO9a-*Trp53*^−/−^-Cas9^+^ cells. Kaplan–Meier survival curves of the mice are shown (n = 4 each group)

To obtain *Trp53*-deficient cells expressing Cas9, we first crossed *Trp53*^−/−^ mice with Cas9 knockin mice. Bone marrow cells derived from 5-FU-treated *Trp53*^*−/−*^-Cas9 or Cas9 mice were transduced with RUNX1-ETO9a (coexpressing Thy1.1), followed by the transplantation of 2 × 10^5^ cells into lethally irradiated (9.5 Gy) Ly5.1 mice (Fig. 1A). All mice receiving RUNX1-ETO9a-transduced *Trp53*^*−/−*^-Cas9 cells (n = 7) developed AML approximately 60 days post-transplantation. In contrast, mice transplanted with RUNX1-ETO9a-transduced Cas9 cells with intact *Trp53* showed considerable variation in disease onset time, with only 5 out of 7 mice developed AML (Fig. 1B). In the diseased mice, bone marrow and spleen were dominated by Thy1.1^+^c-Kit^+^ immature blasts, with a trend that *Trp53* deficiency increased the c-Kit^+^ immature cells (Fig. 1C, D).

To confirm the leukemogenic potential of the RUNX1-ETO9a-expressing cells, we then transplanted 1 × 10^6^ spleen cells collected from the moribund primary recipient mice into sublethally irradiated (5.25 Gy) secondary recipient mice. Again, mice transplanted with RUNX1-ETO9a-*Trp53*^*−/−*^-Cas9^+^ cells developed AML more rapidly than those receiving RUNX1-ETO9a-Cas9^+^ cells with intact *Trp53* (Fig. 1E). We observed the accumulation of c-Kit^+^ cells in the spleens of the secondary recipient mice, similar to the primary recipient mice (Fig. 1F). Thus, RUNX1-ETO9a alone can initiate AML development, and its combination with *Trp53* deficiency significantly accelerates disease progression.

We then enriched leukemia stem cell activity of these RUNX1-ETO9a cells through tertiary and quaternary transplantation, finally resulting in the generation of aggressive AML cells capable of producing leukemia in 10 days even in non-irradiated recipient mice (Fig. 1G).

### Distinct impact of RUNX1 depletion on *Trp53*-intact or deficient RUNX1-ETO9a cells

**Fig. 2:**
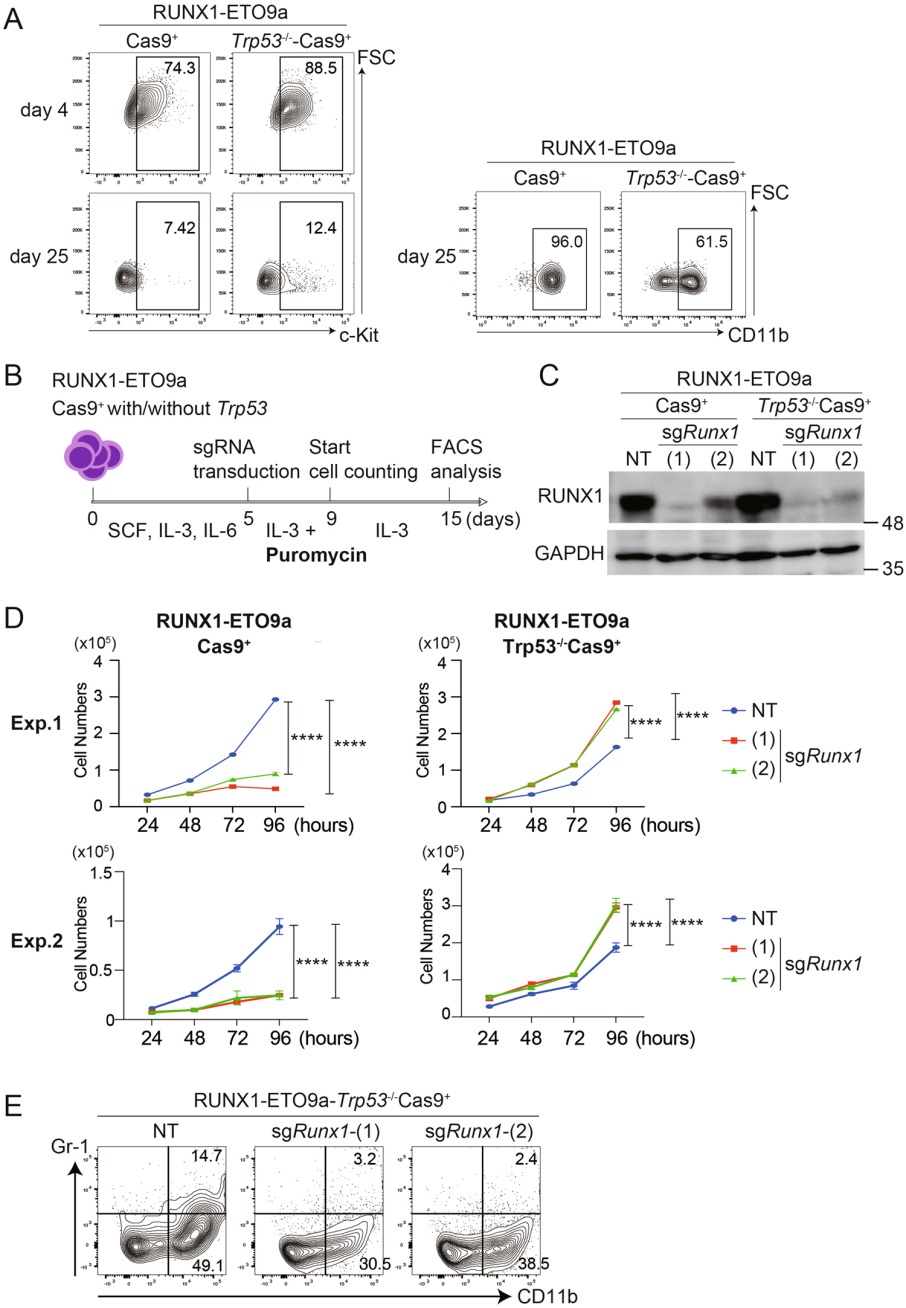
Opposing effects of *Runx1* depletion in *Trp53*-intact or deficient t(8;21) AML. **A** RUNX1-ETO9a-Cas9^+^ and RUNX1-ETO9a-*Trp53*^−/−^-Cas9^+^ cells were cultured and evaluated with markers for primitive (c-Kit) and differentiated (CD11b) cells at day 4 and day 25. **B** Scheme of the experiments used in (**C**–**E**). RUNX1-ETO9a-Cas9^+^ and RUNX1-ETO9a-*Trp53*^−/−^-Cas9^+^ cells were transduced with non-targeting (NT) sgRNA or sgRNAs targeting mouse *Runx1* [sg*Runx1*-(1) and (2)]. Some cells were subjected to western blotting (**C**) and the remaining cells were cultured for 96 h to compare the growth (**D**) and differentiation (**E**). **C** Levels of RUNX1 and GAPDH protein in RUNX1-ETO9a-Cas9^+^ and RUNX1-ETO9a-*Trp53*^−/−^-Cas9^+^ cells transduced with NT or *Runx1*-targeting sgRNAs. **D** The numbers of the cells were evaluated every 24 h. Results are shown as means ± SD of three experiments. ****P < 0.0001. Two independent experiments (Exp.1 and Exp.2) using different clones were performed. **E**. Expression of CD11b and Gr-1 (markers for myeloid maturation) was evaluated in NT or sg*Runx1*-(1)/(2)-transduced RUNX1-ETO9a-*Trp53*^−/−^-Cas9^+^ cells

Next, we examined if the RUNX1-ETO9a cells can be cultured in vitro. We first cultured the RUNX1-ETO9a-*Trp53*^*−/−*^-Cas9^+^ cells and RUNX1-ETO9a-Cas9^+^ cells obtained from moribund mice in RPMI-1640 medium with murine SCF, IL-3, and IL-6 for 5 days, and then reduced the concentrations of SCF and IL-6 gradually. The RUNX1-ETO9a cells with/without *Trp53* deficiency grew well in the medium containing only IL-3 for at least 2 weeks. However, we observed a significant decline in the proliferative capacity of cells after approximately 20 days of in vitro culture. Both the *Trp53*-intact or deficient RUNX1-ETO9a cells were differentiated into mature myeloid cells, as evidenced by the reduced c-Kit expression and a remarkable increase of CD11b^+^ cells at day 25 (Fig. 2A). Thus, RUNX1-ETO9a cells were not immortalized in vitro, but could be cultured for up to three weeks, which is sufficient for most in vitro experiments.

We then assessed the effect of RUNX1 depletion on the growth of *Trp53*-intact/deficient RUNX1-ETO9a cells. RUNX1 has been shown to promote the efficient growth of AML cells including t(8;21) AML, but a previous study reported that RUNX1 promotes the tumor growth only in tumors with intact TP53 [19]. The *Trp53*-intact and deficient RUNX1-ETO9a cells were transduced with non-targeting (NT) or mouse *Runx1*-targeting [sg*Runx1*-1(1) and (2)] sgRNAs and the sgRNA-transduced cells were selected using puromycin for 4 days (Fig. 2B). The efficient depletion of RUNX1 following the introduction of the *Runx1*-targeting sgRNAs was confirmed by Western blotting (Fig. 2C). Interestingly, RUNX1 depletion showed contrasting effects in *Trp53*-intact and *Trp53*-deficient RUNX1-ETO9a cells. Consistent with many previous reports [19–22], RUNX1 depletion showed the strong growth-inhibitory effect in RUNX1-ETO9a-Cas9^+^ cells. In sharp contrast, RUNX1 depletion promoted the growth of RUNX1-ETO9a-*Trp53*^*−/−*^-Cas9^+^ cells by inhibiting their myeloid maturation (Fig. 2D, E). These results highlight the importance of *Trp53* status in determining the effect of RUNX1 depletion in t(8;21) AML.

### *Trp53*-intact or deficient RUNX1-ETO9a cells show distinct drug susceptibility

**Fig. 3:**
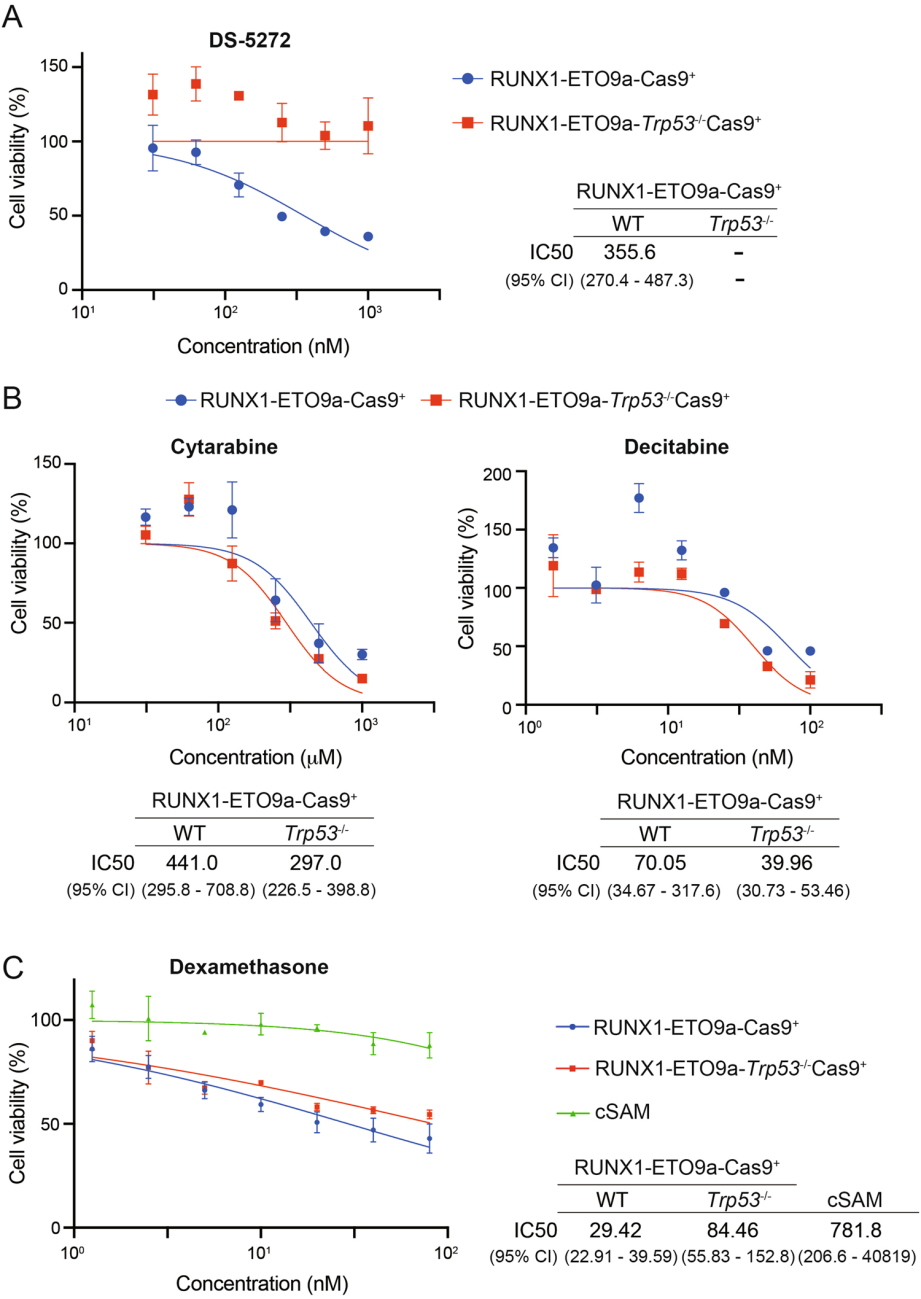
Drug sensitivity of *Trp53*-intact or deficient t(8;21) AML cells.** A**, **B** RUNX1-ETO9a-Cas9^+^ and RUNX1-ETO9a-*Trp53*^−/−^-Cas9^+^ cells were incubated with DS-5272 (**A**), cytarabine, or decitabine (**B**) at the indicated concentration for 72 h. Cell viability was evaluated with the Cell Counting Kit-8. Data are shown as means ± SD from three technical replicates. **C** RUNX1-ETO9a-Cas9^+^ cells, RUNX1-ETO9a-*Trp53*^−/−^-Cas9^+^ cells and cSAM cells were incubated with dexamethasone for 72 h. Cell viability was evaluated with the Cell Counting Kit-8. Results are shown as means ± SD from three technical replicates. *WT* wild-type, *CI* confidence Interval

Finally, we assessed the effect of several drugs on the growth of *Trp53*-intact or deficient RUNX1-ETO9a cells using the cell viability assay. As expected, DS-5272, an inhibitor of p53-MDM2 interaction [23, 24], showed the growth-inhibitory effect only in the *Trp53*-intact RUNX1-ETO9a cells (Fig. 3A). We then examined whether *Trp53* status affects the sensitivity of RUNX1-ETO9a cells to common chemotherapeutic agents: cytarabine [2], decitabine [25] and dexamethasone [26]. Although *Trp53* deletion did not alter the sensitivity of RUNX1-ETO9a cells to these drugs, we found that RUNX1-ETO9a cells were particularly sensitive to dexamethasone regardless of *Trp53* status even at low concentrations (Fig. 3B, C). In contrast, cSAM, another murine AML cell line transformed by SETBP1-D868N and ASXL1-E635RfsX15 mutations [12, 13], was resistant to dexamethasone treatment (Fig. 3C). Thus, dexamethasone was specifically effective against t(8;21) AML, including those with *TP53* alterations.

## Discussion

Although t(8;21) AML has been classified as a favorable risk AML, a significant proportion of patients, especially those with specific co-operating mutations, often relapse and eventually die. *TP53* is one of the genes whose mutations are associated with poor prognosis in t(8;21) AML [5]. In this study, we established a novel murine model for t(8;21) AML with/without *Trp53* deficiency using RUNX1-ETO9a, a short isoform of RUNX1-ETO with stronger leukemogenic potential [9]. The RUNX1-ETO9a cells are able to generate AML in vivo and can be cultured in vitro for up to three weeks. In addition, the RUNX1-ETO9a cells established in this study express Cas9. Therefore, any gene of interest can be efficiently depleted in these cells.

Previous experimental studies have shown that loss of TP53 promotes disease progression and therapy resistance in RUNX1-ETO leukemia [7, 8]. Consistent with these findings, we showed that *Trp53* deficiency accelerates the development of AML driven by RUNX1-ETO9a. However, it should be noted that *Trp53* was already deleted in cells prior to RUNX1-ETO9a transduction in all these *Trp53*-deficient t(8;21) AML models. Given that *TP53* mutations are typically detected as secondary somatic mutations in t(8;21) AML, and that acute and chronic inhibition of TP53 sometimes show opposing effects [27], the effect of late *Trp53* depletion in the established RUNX1-ETO9a leukemia warrants further investigation. The Cas9^+^RUNX1-ETO9a cells established in this study will be useful for this purpose. Furthermore, our mouse t(8;21) AML models will provide ideal platforms to perform the in vivo CRISPR/Cas9 library screening to identify key regulators that promote or suppress the development of RUNX1-ETO leukemia, particularly in vivo.

Using these RUNX1-ETO9a cells with or without *Trp53* deficiency, we showed that targeting RUNX1 is only effective in *Trp53*-intact RUNX1-ETO9a cells. Previous studies have shown that RUNX1 has a dual role in leukemogenesis [28]. RUNX1 acts as a tumor promoter by promoting the survival of AML cells [20, 21], in part through activation of TP53-mediated pro-apoptotic signaling [19, 29]. On the other hand, RUNX1 also acts as a tumor suppressor by inhibiting myeloid maturation [20, 30]. Therefore, it is likely that the tumor suppressor role of RUNX1 is more pronounced in the *Trp53*-deficient RUNX1-ETO cells. Thus, our data together with previous findings strongly suggest that the antileukemic effect mediated by RUNX1 depletion requires functional TP53.

Various novel therapeutic strategies for treating RUNX1-ETO leukemia have demonstrated promise in either clinical or experimental investigations [1]. These include a KIT inhibitor dasatinib [31], JAK inhibitors [15, 32], HDAC inhibitors [33], and glucocorticoid drugs such as dexamethasone. In this study, we found that RUNX1-ETO9a cells were particularly sensitive to dexamethasone regardless of *Trp53* status. While glucocorticoids are widely used to treat lymphoid malignancies [26], they are generally not deemed beneficial in the context of AML. However, several previous reports have repeatedly shown that glucocorticoids are effective in suppressing the growth of t(8;21) AML cells at low doses, but not in other subtypes of AML [34, 35]. These findings, together with our data, provide a rational basis for clinical testing of glucocorticoid drugs, such as dexamethasone, against t(8;21) AML including those with *TP53* alterations.

In summary, we established novel murine Cas9^+^ RUNX1-ETO9a cells with intact or deficient *Trp53*. These cells allow testing the effect of novel drugs in vitro and in vivo, enable genetic screens using sgRNA libraries, and will provide valuable information on the role of TP53 in the development of t(8;21) AML in future studies.

## Data Availability

All data will be made available on reasonable request.
